# The contribution of DNA repair pathways to *Staphylococcus aureus* fitness and fidelity during nitric oxide stress

**DOI:** 10.1128/mbio.02156-23

**Published:** 2023-11-10

**Authors:** Kelly E. Hurley, Srijon K. Banerjee, Amelia C. Stephens, Michelle R. Scribner, Vaughn S. Cooper, Anthony R. Richardson

**Affiliations:** 1 Department of Microbiology and Molecular Genetics, University of Pittsburgh, Pittsburgh, Pennsylvania, USA; New York University School of Medicine, New York, New York, USA; University of Toronto, Toronto, Canada

**Keywords:** DNA repair, *Staphylococcus aureus*, nitric oxide

## Abstract

*Staphylococcus aureus* is a major human pathogen that causes a variety of illnesses, ranging from minor skin and soft tissue infections to more severe systemic infections. Although the primary host immune response can typically clear bacterial infections, *S. aureus* is uniquely resistant to inflammation. For instance, our laboratory has determined that *S. aureus* is highly resistant to nitric oxide (NO⋅), an important component of the innate immune response that plays a role in both immunomodulatory and antibacterial processes. Additionally, NO⋅ and its derivatives can cause damage to *S. aureus* DNA, more specifically, deamination and/or oxidation of DNA bases; however, regulation and repair mechanisms of DNA in *S. aureus* are understudied. Thus, we hypothesize that several DNA repair mechanisms may account for the replication fidelity of *S. aureus* and may contribute to fitness in the presence of NO⋅. Here, we show the role of several DNA repair mechanisms in *S. aureus*. More specifically, we found that recombinational repair genes *recJ*, *recG*, and *polA* may play a role in the repair of NO⋅-induced replication fork collapses. We also show the role of the base excision repair pathway protein, MutY, in reducing NO⋅-mediated mutagenesis. Overall, our results suggest that NO⋅ leads to DNA damage, which subsequently induces the activity of several DNA repair pathways, contributing to the replication fidelity and fitness of *S. aureus*.

**IMPORTANCE:**

Pathogenic bacteria must evolve various mechanisms in order to evade the host immune response that they are infecting. One aspect of the primary host immune response to an infection is the production of an inflammatory effector component, nitric oxide (NO⋅). *Staphylococcus aureus* has uniquely evolved a diverse array of strategies to circumvent the inhibitory activity of nitric oxide. One such mechanism by which *S. aureus* has evolved allows the pathogen to survive and maintain its genomic integrity in this environment. For instance, here, our results suggest that *S. aureus* employs several DNA repair pathways to ensure replicative fitness and fidelity under NO⋅ stress. Thus, our study presents evidence of an additional strategy that allows *S. aureus* to evade the cytotoxic effects of host NO⋅.

## INTRODUCTION

*Staphylococcus aureus* is a Gram-positive bacterium that is a highly invasive human pathogen. It is known to cause a variety of illnesses, ranging from superficial skin and soft tissue infections to more severe systemic infections such as endocarditis, osteomyelitis, and sepsis (1). *S. aureus* asymptomatically colonizes the anterior nares and skin, and an estimated 30% of the human population are natural carriers (2). The treatment of infections has become increasingly difficult due to antibiotic resistance. As such, methicillin-resistant *Staphylococcus aureus* (MRSA) has been the most common cause of infection since the 1960s (3). Historically, it is known that MRSA-related hospitalizations lead to severe morbidity and mortality globally. However, in recent decades, community-acquired MRSA (CA-MRSA) infections have been increasingly found in otherwise healthy populations (4). Additionally, CA-MRSA clones have been found to be phylogenetically distinct from hospital-associated MRSA and have exhibited both hypervirulence and improved transmission within the host.

One major factor contributing to *S. aureus* pathogenesis is resistance to the broad-spectrum antimicrobial immune radical, nitric oxide (NO⋅). NO· is an important component of the host innate immune response and plays a role in antibacterial and immunomodulatory processes (5). Although inflammatory NO⋅ is typically required for clearance of pathogenic bacterial infections, *S. aureus* is uniquely resistant to this immune radical, which distinguishes *S. aureus* from closely related coagulase-negative staphylococcal species that are unable to grow in its presence (6). During infection, NO⋅ is produced by activated phagocytes through the inducible nitric oxide synthase (iNOS) and can react directly with invading organisms in surrounding inflamed host tissues (7, 8). NO⋅ and its derivatives are known to target heme, iron-sulfur clusters, thiols, lipids, and DNA (9–11). Accordingly, under physiologically relevant concentrations of NO⋅, the reversible binding of NO⋅ to cytochrome heme centers results in the inhibition of aerobic respiration, which can be restored once NO⋅ is detoxified (12). The reactions of NO⋅ and its targets subsequently interfere with many pathways, inducing metabolic and replicative stress. *S. aureus* therefore must evolve mechanisms of survival under these conditions. The mechanism underlying *S. aureus* resistance to NO⋅ is complex and consists of several metabolically regulated gene products (5, 6, 13). With that said, we are still lacking in understanding of what makes *S. aureus* effective resistance to NO⋅ so unique. As previously stated, it is known that NO⋅ targets *S. aureus* DNA, leading to DNA damage (9, 10). More specifically, NO⋅ leads to the oxidation and deamination of DNA bases; however, the regulation and repair mechanisms of DNA in *S. aureus* are understudied.

In several bacteria, DNA is damaged due to several endogenous and exogenous factors such as radiation, chemicals, and environmental stress (14). This damage can, in turn, inhibit replication and downstream gene transcription, ultimately affecting cell survival and leading to the accumulation of mutations (15). DNA damage can result from replication fork collapses, single-strand breaks, or exposure to metabolic byproducts such as reactive oxygen species (ROS) or reactive nitrogen species (RNS) (16). Since *S. aureus* has uniquely evolved to replicate in the presence of NO⋅, we wanted to determine if there were any DNA repair pathways that enhanced the overall fitness of this pathogen in the host environment as well as contributed to replication integrity under NO⋅ stress. We know that NO⋅ exposure results in deamination and/or oxidation of DNA bases, but the regulation and repair mechanisms of this damage are unknown in *S. aureus*. Thus, we employed mutants from the nucleotide excision repair (NER) (e.g., *uvrABC*), base excision repair (BER) (e.g., *nth*, *nfo*, *mutY*, and *ung*), recombination repair (e.g., *queA*, *topB*, and *sbcC*), and replication fork restart pathways (e.g., *polA*, *recG*, *recJ*, and *recQ*) to determine a role under NO⋅ stress. Overall, DNA repair mechanisms may pose a target for novel therapeutics that sensitize pathogens to effectors of the host defense.

## RESULTS

### Mutation accumulation analysis suggests that NO⋅ stress leads to deamination and/or oxidation of DNA in *S. aureus*

Since we speculate that NO⋅ targets DNA and leads to deamination and/or oxidation of *S. aureus* DNA bases, we performed a modified mutation accumulation assay to determine if there were any specific mutations accumulated under NO⋅ stress compared to an unexposed group (17). We passaged 40 lineages for 40 days, both in the presence and absence of NO·. Upon alignment of whole genome sequencing reads to a reference genome, we found that NO· exposure increased the number of accumulated mutations by more than 25%. That is, 173 mutations were in the exposed group, and 112 were in the unexposed group (Table 1). Additionally, as with many bacteria, we found that 70% of the mutations in both groups resulted in GC→AT mutations, potentially explaining the fact that the *S. aureus* genome is nearly 70% AT. The mutations most frequently induced under NO⋅ stress compared to the unexposed group were C:G→T:A, G:C→A:T, C:G→A:T, and T:A→C:G, which are all products of DNA deamination and/or oxidation. Another, more rare form of mutation, tandem base substitutions, only occurred in NO·-exposed lineages (Table 1). Tandem base substitutions are thought to arise through the deamination of cytosine to uracil in DNA, followed by subsequent excision by Ung. Other rare mutations that occurred in the untreated strains were mutation 10 (Table 1), which was a loss of a small 3.125 kb plasmid, and mutation 13 (Table 1), which was the loss of the ACME and SCC-Mec islands. Together, these findings confirm that NO⋅ exposure results in the deamination and oxidation of DNA bases in *S. aureus*, primarily cytosine and guanine.

**TABLE 1:** Mutation accumulation

Mutation	Group − NO⋅	Group + NO⋅	Total
(A)7→6	1	0	1
(A)7→8	2	0	2
(ATT)7→6	1	1	2
(T)7→6	1	0	1
(T)7→8	4	1	5
(TACAGAAACAAA)2→1	0	1	1
2 bp→AC	0	1	1
2 bp→AG	0	1	1
2 bp→TT	0	1	1
Δ3,125 bp	2	0	2
Δ4 bp	1	0	1
Δ54 bp	0	1	1
Δ54,659 bp	1	0	1
Δ69 bp × 2	1	1	2
A→C	1	5	6
A→G	11	9	20
A→T	5	8	13
C→A	6	23	29
C→G	23	22	45
C→T	39	68	107
G→A	75	88	163
G→C	2	4	6
G→T	22	9	31
T→A	4	5	9
T→C	4	12	16
T→G	3	2	5
	209	263	472

### Identification of DNA repair mechanisms that contribute to *S. aureus* control of NO⋅-induced mutagenesis

**FIG 1:**
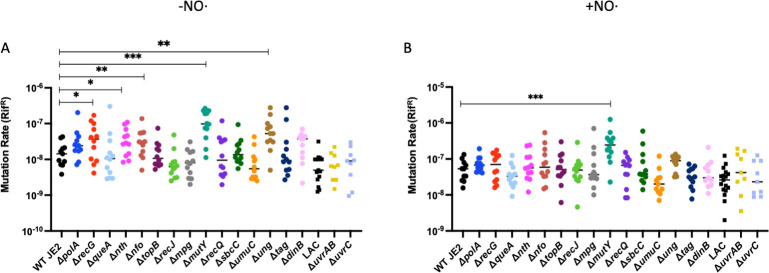
Elevated mutation rates suggest that MutY may play a role in modulating mutagenicity in the presence of NO⋅ in *S. aureus*. Mutability of *S. aureus* WT JE2 and 15 DNA repair transposon mutants shown either unexposed (**A**) or exposed (**B**) to a disc of 500 mM diethylenetriamine NONOate ( (*n* = 12). Data were analyzed via the Wilcoxon rank-sum test for nonparametric analyses (*, *P* < 0.05; **, *P* < 0.005; ***, *P* < 0.0005).

Since we have confirmed that NO⋅ targets *S. aureus* DNA, resulting in deamination and oxidation of DNA bases, we also wanted to determine if there were any DNA repair mechanisms involved in targeting NO⋅-mediated mutagenesis. To do so, we employed 15 DNA repair mutant strains from various DNA repair pathways: NER, BER, recombination repair, and replication fork restart. These specific strains were selected due to the recent identification of the target genes in *S. aureus* based on known homology in *Bacillus subtilis*. We performed mutation rate assays with the 15 DNA repair mutant strains alongside a WT JE2 control. We found that in the absence of NO⋅, Δ*recG*, Δ*nth*, Δ*nfo*, Δ*ung*, and Δ*mutY* transposon mutants display significantly increased mutation rates compared to WT JE2 (Fig. 1A). Upon addition of NO·, the mutation rate of wild type (WT) is elevated by approximately fivefold (*P* = 0.001, Mann-Whitney), consistent with the mutation accumulation results (Table 1). However, the mutation rate of the Δ*mutY* mutant was further increased in the presence of NO⋅, suggesting elevated levels of guanine oxidation (Fig. 1B). Overall, the hypermutable phenotype we observed with the Δ*mutY* mutant under NO⋅ exposure suggests that MutY likely targets NO⋅-mediated mutagenesis in *S. aureus*. Finally, all hypermutability in the ∆*mutY* mutant, either in the absence or presence of NO·, was resolved upon genetic complementation (Fig. S2A and B).

### Identification of DNA repair mechanisms associated with maintaining replicative fitness under NO⋅ stress in *S. aureus*

**FIG 2:**
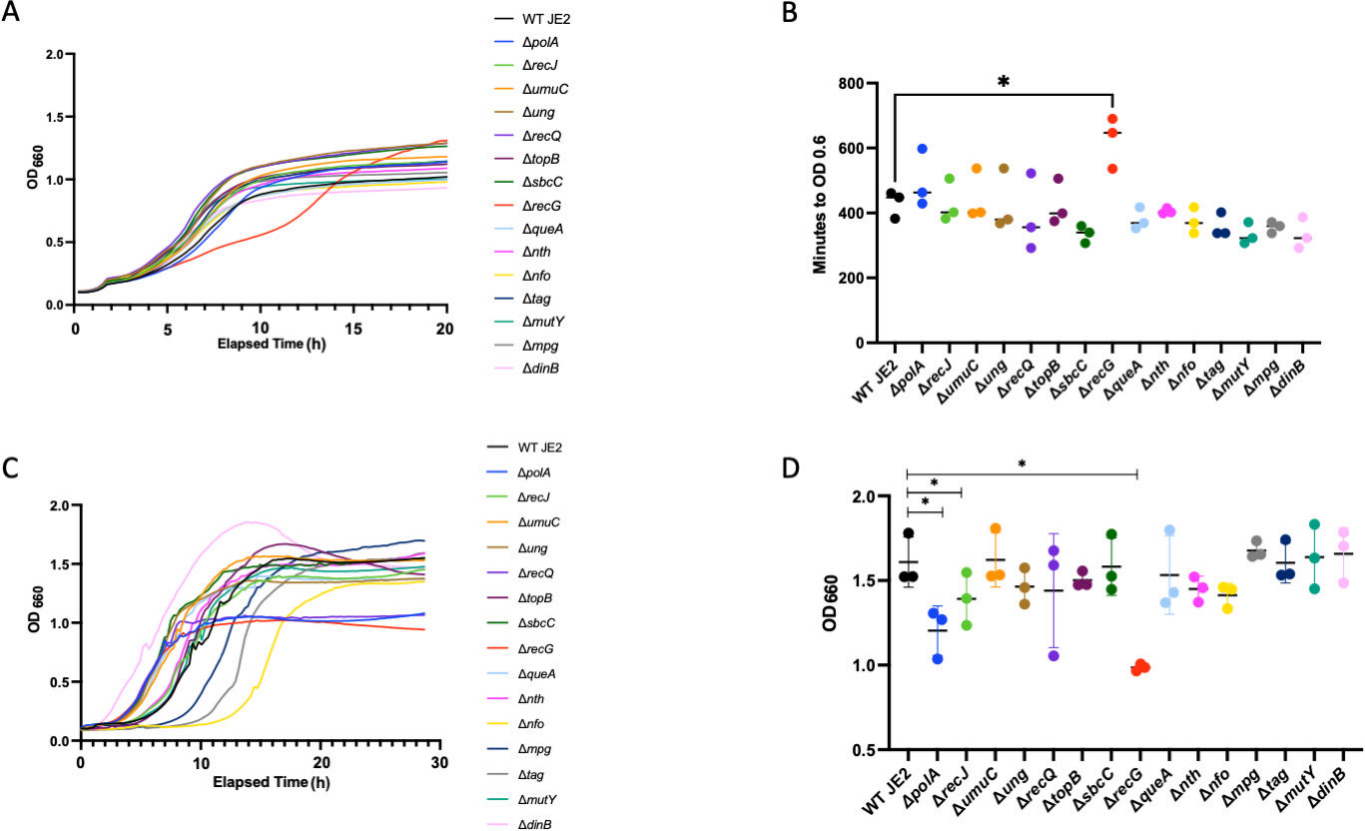
Growth curves suggest that RecG may contribute to *S. aureus* ability to confer fitness under NO⋅ stress. A representative growth curve is shown for *S. aureus* WT JE2 and 15 DNA repair transposon mutants grown in tryptic soy broth with a mixture of 1 mM DEANO and 10 mM NOC-12 added at OD 0.2 (**A**) (*n* = 3). The amount of time it took each mutant strain to reach an OD of 0.6 with a mixture of 1 mM DEANO and 10 mM NOC-12 added at OD 0.2 (**B**) (*n* = 3). A representative growth curve is shown for *S. aureus* WT JE2 and 15 DNA repair transposon mutants grown in PNG with the addition of 10 mM DETA/NO at inoculum (**C**) (*n* = 3). Average terminal OD from growth curves (**C**) of *S. aureus* WT JE2 and 15 DNA repair transposon mutants grown in PNG minimal media with 10 mM DETA/NO added at inoculum (**D**) (*n* = 3). Data were analyzed via one-way analysis of variance with Dunnett’s multiple comparisons test for correction (*, *P* < 0.05) or Student’s two-tailed *t*-test (*, *P* < 0.05) where appropriate.

Although we have identified a DNA repair pathway responsible for controlling NO⋅-induced mutagenesis, we also wanted to determine if there were any DNA repair mechanisms that contribute to replicative fitness under NO⋅ stress. To do so, we employed the same 15 DNA repair mutant strains screened in the previous section alongside a WT JE2 control and performed growth curves. Without the addition of NO⋅, we observed a similar growth rate across all strains (Fig. S1A). However, following the addition of a mixture of NO⋅ donors (1 mM diethylamine NONOate [DEANO] and 10 mM NOC-12) at an OD_660_ of 0.2, one of these strains, Δ*recG*, exhibited a significant growth defect (Fig. 2A and B). Additionally, we performed growth curves in chemically defined media, exposed the cultures to a NO⋅ donor [10 mM diethylenetriamine NONOate (DETA/NO)] at inoculum, and observed average terminal optical density (OD) across all DNA repair mutant strains (Fig. 2C). The results show a significantly lower average terminal OD for Δ*polA*, Δ*recJ*, and Δ*recG* than for WT JE2 (Fig. 2D). None of these mutants had a defect in reaching WT terminal OD in the absence of NO· (Fig. S1B). We also observed increased lag times for ∆*nfo*, ∆*mpg*, and ∆*tag* in PN medium; however, none of these mutants showed reduced growth rate or terminal OD (Fig. 2C and D). Taken together, these results suggest that RecG and, to a lesser extent, PolA and RecJ may play roles in maintaining fitness in *S. aureus* in the presence of NO⋅. Finally, the major NO· sensitivity phenotype associated with the ∆*recG* mutation was resolved upon genetic complementation (Fig. S3).

### Replication fork restart DNA repair pathway contributes to NO⋅ resistance *in vivo*

**FIG 3:**
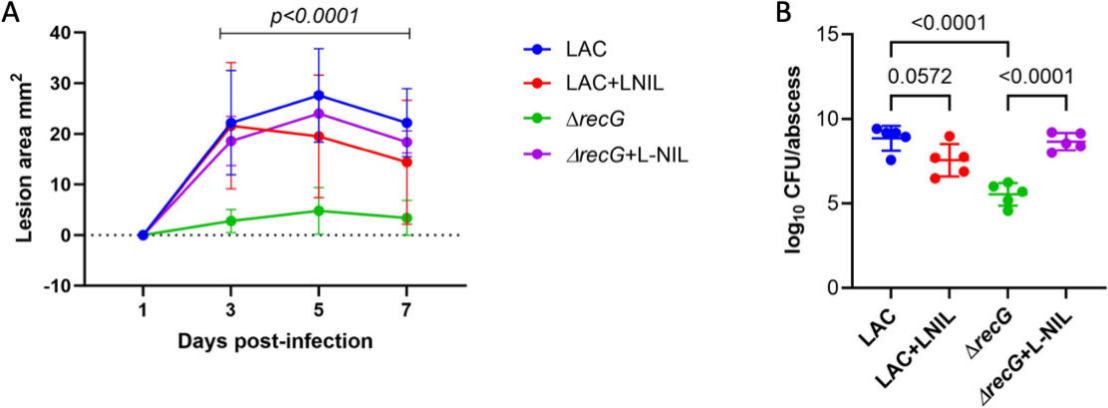
*In vivo* results suggest that RecG plays a role in maintaining replication integrity in *S. aureus* in the presence of host NO⋅ production. C57BL/6J mice were infected subcutaneously with 10^7^ CFU of either WT LAC or the ΔrecG mutant (*n* = 5). A representative graph of lesion sizes measured on days 1, 3, 5, and 7 post infection is shown in A. Bacterial burden was measured by harvesting the abscess 7 days post infection and enumerating the bacteria (**B**). Data were analyzed via one-way analysis of variance with Tukey’s multiple comparisons test for correction.

Since the ∆*recG* mutant exhibited the most consistent and most profound fitness defect in the presence of NO· *in vitro*, we decided to test whether it had a fitness defect due to NO· *in vivo*. In order to confirm the role of RecG in maintaining replication integrity in the presence of NO⋅ *in vivo*, we infected C57BL/6J mice subcutaneously with 10^7^ colony-forming units (CFU) of either *S. aureus* WT LAC or *S. aureus* Δ*recG* mutant. The mice were assessed both 3 and 7 days post infection by monitoring lesion size and bacterial burden within the abscess. Our results show that mice infected with the Δ*recG* mutant display a significant decrease in both lesion size and bacterial burden in infected abscesses compared to WT-infected mice (Fig. 3A and B). This suggests that mice infected with the Δ*recG* mutant develop a less severe infection than those infected with WT *S. aureus*. Additionally, to determine the direct role of host NO⋅ production during infection, we treated mice with an iNOS inhibitor, N-iminoethyl-L-lysine (L-NIL), and observed the effect on Δ*recG* mutant-infected mice. The L-NIL-treated mice infected with the Δ*recG* mutant displayed a lesion size and bacterial burden in infected abscesses similar to WT levels, which were significantly elevated compared to untreated Δ*recG* mutant-infected mice. While L-NIL-treated animals can still generate ROS, the ∆*recG* mutant does not display a severe growth defect in the presence of H_2_O_2_ when compared with WT, suggesting that NO· specifically necessitates RecG during infection (Fig. S4). Overall, our results suggest a role for RecG in protecting against *S. aureus* cell death due to host NO⋅ production during infection.

## DISCUSSION

In response to a typical *S. aureus* infection, activated host innate immune cells produce reactive nitrogen species that attack various targets of the cell. The most direct targets are enzymes in the respiratory chain, the blocking of which leads to increased intracellular reactive oxygen species (18). Since we know that RNS and ROS target *S. aureus* DNA, likely resulting in deamination and/or oxidation of DNA bases, we performed a mutation accumulation (MA) experiment to observe via sequencing analysis which mutations most frequently occur under NO⋅ stress. The MA experiment is ideal to estimate rates of spontaneous mutations that occur over the duration of the experiment, and in our case, we were able to compare the accumulation of mutations under NO⋅ exposure compared to an unexposed group (17). Additionally, we performed our experiment without selection, ensuring the elimination of selection bias on mutant frequency. Overall, we observed that the mutations that were most frequently induced by NO⋅ were all products of DNA oxidation and deamination: C→T, G→A, C→A, and T→C (19). The mutations that occurred most frequently under NO⋅ stress were C→T and G→A transitions. These mutations likely occurred due to the deamination of cytosine or guanine (9, 20). A C→T transition can arise in two mechanisms. First, deaminating guanine to xanthine will result in a C:X pair. Since xanthine typically pairs with thymine, upon further replication, the C:X would result in a T:X pair and finally be repaired to T:A. The second mechanism involving a C:G pair under NO⋅ exposure is that the cytosine is deaminated to uracil, which results in a U:G pair, followed by synthesis to a U:A pair, and upon further replication, results in a T:A pair. Another result of cytosine deamination followed by the excision of uracil by Ung is tandem base substitutions (21). This is due to the tendency of dU bases to “flip” out of the double helix. Following a round of replication and subsequent excision of uracil, two tandem base mutations can occur. Indeed, all three tandem base substitutions were detected in the NO·-treated lineages and all derived from parental C:G sites (Table 1). The C→A transversion occurred less frequently; however, it is interesting due to the bias of occurrence in the NO⋅ exposed group. A common DNA base lesion formed during replication stress is 8-oxoG, which can mismatch to adenine (22). What most likely occurs is a C:G pair oxidized to C:8-oxoG, followed by replication to an A:8-oxoG mispair. Upon replication, A:oxo-G is likely repaired to A:T, resulting in a C→A transversion. Finally, the mutation that least likely occurred is the T→C transition. This transition likely arises due to the deamination of adenine to hypoxanthine, so a T:A pair turns into T:HX. HX typically binds to cytosine, resulting in a HX:C pair, which, following another round of replication, results in a C:G pair. Ultimately, our results show that NO⋅ exposure results in the accumulation of DNA transitions over transversions. Furthermore, the overall takeaway from the MA analysis is that the exposure of WT *S. aureus* to NO⋅ results in accumulation of DNA damage, more specifically, deaminated and oxidized DNA. *S. aureus* likely relies on the BER pathway to repair these accumulated lesions, ultimately aiding the overall ability of this pathogen to modulate mutagenesis in a NO⋅-concentrated environment.

Though we have confirmed that NO⋅ exposure results in deamination and oxidation of DNA bases in *S. aureus*, we also wanted to know if there were any DNA repair mechanisms contributing to *S. aureus* ability to mitigate mutagenesis in this NO⋅-concentrated environment. We observed mutagenicity across several DNA repair transposon insertion mutants compared to WT JE2, both in the presence and absence of NO⋅. Overall, we observed increased mutation rates in Δ*recG*, Δ*nth*, Δ*nfo*, Δ*mutY*, and Δ*ung* compared to WT in the absence of NO⋅, suggesting that inactivation of these genes results in the acquisition of spontaneous mutations. RecG is an ATP-dependent helicase involved in replication fork restart following double-strand breaks (16). Without RecG activity, we would expect to see an increase in mutation rate since its absence would likely result in the incomplete resolution of DNA Holliday junctions and, thus, an accumulation of mutagenic DNA. Nth, Nfo, MutY, and Ung are all members of the BER pathway involved in single-stranded DNA damage repair (23). The main function of the BER pathway is to repair nonbulky single-base DNA lesions such as oxidized or deaminated DNA bases, alkylated or abasic sites, and dUTP incorporation during DNA replication (24). The misincorporation of dUTP during DNA replication likely occurs one of two ways: either due to deamination of cytosine residues in DNA or incorporation of dUTP before the intermediate can be catalyzed to dTTP, since the replicative polymerase cannot distinguish between them (25, 26). Nth and Nfo are endonuclease III and endonuclease IV, respectively. They both play a role in resolving apurinic or apyrimidic (AP) sites left by DNA glycosylase activity when repairing damaged DNA during BER (27, 28). In inactivating these genes, we would expect to see the increase in mutability that we observe in our results since insufficient removal of the AP site would subsequently result in an interruption of this repair process. Ung and MutY are both DNA glycosylases involved in BER (29, 30). Ung excises uracil residues from DNA. MutY is an adenosine DNA glycosylase that hydrolyzes free adenine bases from 8-oxo-guanine:A mismatches (31, 32). An inactivated *mutY* or *ung* mutant strain would likely lack the capability to initiate the BER process, resulting in the accumulation of single-base lesions in DNA left unresolved, which likely explains our results. Interestingly, following the addition of NO⋅ in our mutation rate assay, we observed that Δ*mutY* displays a further enhanced mutation rate. Since we know that NO⋅ causes deamination and/or oxidation of DNA bases and both Ung and MutY target oxidized and/or deaminated DNA bases, the hypermutable phenotype seen with the inactivated Δ*mutY* strain is likely a result of the accumulation of unresolved oxidized DNA bases (33). Overall, our results suggest a role of the BER pathway, more specifically, the DNA glycosylase MutY, in targeting NO⋅-mediated mutagenesis in *S. aureus*.

Although we have identified a process involved in controlling NO⋅ stress-induced mutagenesis in *S. aureus*, we wanted to determine if there were any DNA repair mechanisms that contribute to replicative fitness under NO⋅ stress. We performed growth curves with a WT JE2 strain alongside several DNA repair transposon insertion mutant strains and compared the optical density over an elapsed time in the presence and absence of NO⋅. Under NO⋅ stress, the Δ*recG* mutant displayed the most significant growth defect, suggesting that this pathway may aid *S. aureus* ability to confer fitness in a NO⋅-stressed environment. This phenotype was further explained by infecting mice with the Δ*recG* mutant, confirming that mice develop a less severe infection than WT-infected mice. However, in mice treated with L-NIL, this phenotype is completely reversed, which suggests that the replication fork restart pathway player, RecG, plays an important role in protecting *S. aureus* from host production of NO⋅ during infection. RecG is an ATP-dependent helicase with 3′–5′ activity and typically works alongside RecJ, which is a single-stranded DNA-specific exonuclease with 5′–3′ activity and also showed a modest growth defect under NO· stress (Fig. 2) (16). Both play a critical role in homologous recombination and DNA repair; more specifically, they play a role in catalyzing branch migration during replication fork restart (34). In the event of DNA double-strand breaks (DSBs) and subsequently a replication fork collapse, homologous recombination is initiated. Typically, the ends of a DSB are processed by a 5′–3′ helicase-nuclease complex, RexAB, which leaves behind a 3′-ssDNA overhang (35). This allows a recombinase, RecA, to bind to the 3′-ssDNA overhang while it searches for a homologous sequence to initiate strand invasion. Following strand invasion, DNA synthesis can occur whereby the 3′ end is extended via DNA polymerase III simultaneously alongside branch migration, carried out by the 3′–5′ helicase, RecG (16). Ultimately, the Holliday junction resolution is carried out by the resolvase protein, RecU. Previously, it has been shown that NO⋅ causes replication fork collapses, which can result in DNA double-strand breaks, leading to mutagenic DNA rearrangement in *S. aureus* (36). In this study, our results suggest a putative role of RecG in maintaining replicative fitness under NO⋅ stress.

MRSA poses a serious threat to population health worldwide. Since *S. aureus* has also uniquely evolved resistance to the primary host immune defense, inflammatory NO⋅, our laboratory sought to determine the mechanisms *S. aureus* employs in order to thwart the activity of NO⋅. In this study, we found several DNA repair mechanisms that enhance *S. aureus* replicative fidelity and overall fitness in this NO⋅-concentrated environment. Thus, these DNA repair mechanisms could potentially act as a target for novel therapeutics that sensitize this pathogen to the primary host immune response. Finding alternative therapies to target this multi-drug-resistant pathogen is essential to circumventing the global burden of antibiotic resistance.

## MATERIALS AND METHODS

### Bacterial strains and growth conditions

*S. aureus* cultures were grown in brain heart infusion (BHI) medium, shaken at 250 rpm at 37°C overnight. *S. aureus* JE2 and LAC USA300 were used in this study as background strains (Table 2). Antibiotic selection in *S. aureus* was carried out using erythromycin (5 µg/mL) where appropriate. All Nebraska Tn mutations were transduced into fresh WT backgrounds and PCR verified prior to analyses. Complementation of the ∆*recG* and ∆*mutY* mutations was performed by cloning each gene and its promoter region into pOS1 to generate strains AR1762 and AR1767, respectively (Table 2).

**TABLE 2:** List of strains and plasmids used in the experiment

Strains	Genotype	Source/reference
*S. aureus* JE2	Methicillin-resistant *S. aureus* laboratory strain	Laboratory strain
*S. aureus* LAC	USA300 methicillin-resistant clinical isolate; laboratory strain	Laboratory strain
NE11	*S. aureus* JE2 ∆*recJ*::erm^R^	(37)
NE22	*S. aureus* JE2 ∆*polA*::erm^R^	(37)
NE152	*S. aureus* JE2 ∆*topB*::erm^R^	(37)
NE445	*S. aureus* JE2 ∆*umuC*::erm^R^	(37)
NE761	*S. aureus* JE2 ∆*nth*::erm^R^	(37)
NE888	*S. aureus* JE2 ∆*ung*::erm^R^	(37)
NE972	*S. aureus* JE2 ∆*recQ*::erm^R^	(37)
NE1028	*S. aureus* JE2 ∆*nfo*::erm^R^	(37)
NE1040	*S. aureus* JE2 ∆*mutY*::erm^R^	(37)
NE1344	*S. aureus* JE2 ∆*recG*::erm^R^	(37)
NE1379	*S. aureus* JE2 ∆*queA*::erm^R^	(37)
NE1451	*S. aureus* JE2 ∆*sbcC*::erm^R^	(37)
NE1613	*S. aureus* JE2 ∆*mpg:*:erm^R^	(37)
NE1825	*S. aureus* JE2 ∆*tag*::erm^R^	(37)
NE1866	*S. aureus* JE2 ∆*dinB*::erm^R^	(37)
AR 1758	*S. aureus* LAC ∆*recG*::erm^R^	This study
AR1762	*S. aureus* LAC ∆*recG*::erm^R^ + pAS12 (pOS1 with *recG*)	This study
AR1767	*S. aureus* JE2 ∆*mutY*::erm^R^ + pAS13 (pOS1 with *mutY*	This study

### Growth rate analysis

*S. aureus* cultures were grown overnight in BHI at 37°C, shaking at 250 rpm. Overnight cultures were washed three times with phosphate-buffered saline (PBS) and inoculated at a 1:200 ratio in a 96-well plate containing tryptic soy broth (200 µL/well). Cells were grown at 37°C and shaken in a BioTek microplate reader. For NO⋅ growth curves, a mixture of 10 mM NOC-12 (t_1/2_ = 100 min) and 1 mM DEANO (t_1/2_ = 2 min) was added at an OD_660_ of 0.20. Growth was monitored every 15 min for 24 h. This provides a rapid and acute NO· stress for a mid-exponential culture to respond to.

### Mutation rate assay

*S. aureus* cultures were shaken at 250 rpm at 37°C overnight. Overnight cultures were serially diluted and plated on BHI agar either with or without exposure to NO⋅. The NO⋅ donor we used in this experiment was DETA/NO, which was resuspended in 0.01 N NaOH and has a half-life (t_1/2_) of 20 h. In cultures exposed to NO⋅, a disc was placed in the center of the plate, and 20 µL of 500 mM DETA/NO was added to the disc. Plates were incubated at 37°C. On the following day, 20 single colonies were picked from both NO⋅-exposed and NO⋅-unexposed plates and resuspended in 200 µL of PBS within a 96-well plate. Each resuspension was serially diluted and subsequently plated on BHI agar plates containing rifampicin (100 µg/mL) and BHI agar plates lacking antibiotics. Following incubation overnight at 37°C, we were able to calculate the mutation rate by dividing the number of colonies found on the BHI + rifampicin plate by the CFU/mL on BHI agar plates lacking antibiotic.

### Mutation accumulation assay

*S. aureus* WT USA300 culture was struck out on 80 tryptic soy agar (TSA) plates. Forty plates were exposed to NO⋅ (500 mM DETA/NO), and 40 plates were unexposed. For 40 consecutive days, a single colony was picked and struck out onto a fresh TSA plate, either with or without exposure to NO⋅. For the NO⋅-exposed plates, a dot was randomly drawn near the NO⋅ disc prior to incubation at 37°C to ensure elimination of selection bias. Finally, on day 40, a single colony was picked and struck out to create a lawn on a fresh TSA plate. On the following day, the lawn was resuspended in 50% BHI and glycerol and stored at −80°C. Genomic DNA was extracted from the 80 isolates and subjected to sequencing analysis.

### Whole genome sequencing analysis

At the end of the mutation accumulation experiment, we extracted genomic DNA from the 80 isolates using the Epicentre MasterPure Gram Positive DNA Purification Kit (Qiagen). DNA was sequenced using Illumina NextSeq 500. We sequenced one clone from the final time point of every evolved lineage. Sequencing reads were trimmed and quality filtered using Trimmomatic version 0.36 with the following criteria: LEADING:3 TRAILING:3 SLIDINGWINDOW:4:15 MINLEN:36 (38). Reads were aligned to a reference genome closely related to the ancestral strain (*Staphylococcus aureus* subsp. *aureus* USA300_FPR3757), and variants were called using breseq version 0.35.0 in consensus mode (39). Breseq was run with default parameters, except that a minimum of five reads from each strand were required to support variant calls. The sample read depth ranged from 91 to 355×. Each lineage was monitored for the possibility of cross-contamination.

### Murine infection model

*S. aureus* LAC and Δ*recG* were grown for 12–16 h in BHI at 37°C. Cultures were diluted to 1:200 in fresh BHI and grown until OD_600_ reached 2.0. Then, 1 mL of each culture was harvested at 10,000 × *g* and washed twice with 1 mL of PBS. Bacterial pellets were reconstituted in 200 µL of PBS and serially diluted to 10^10^. All dilutions were plated on BHI agar. Colonies were counted the next day, while the bacterial suspensions were stored at 4°C. Bacterial suspensions were adjusted to 5 × 10^8^/mL based on CFU enumeration. Both male and female, 6–8-week-old C57BL/6J mice weighing 20–25 g were used in this study. Mice were obtained from Jackson Laboratories (Bar Harbor, ME, USA) and housed with 14-h light cycles. On the day of infection, mice were weighed, and 12× (body weight) in µL of Avertin/2,2,2-tribromoethanol (Acros) was administered via intraperitoneal injection. The left flank on the dorsal side of each animal was shaved, and 20 µL or 10^7^ CFU of the bacterial suspension was injected subcutaneously using a 26G needle. Animals were monitored every day, and abscesses were measured on days 3, 5, and 7. On day 7, the mice were euthanized in a CO_2_ chamber, followed by cervical dislocation. The abscesses were excised precisely, avoiding extra tissue, and homogenized in PBS. The homogenates were serially diluted and plated on BHI agar for CFU enumeration the next day. For L-NIL treatment, 100 µg/mL of L-NIL hydrochloride (Cayman) was added to the drinking water 24 h prior to infection. The L-NIL water was changed every 2 days from the day of infection.

## SUPPLEMENTAL MATERIAL

Figure S1 mbio.02156-23-s0001.tif Growth without NO.

Figure S2 mbio.02156-23-s0002.tif MutY complementation.

Figure S3 mbio.02156-23-s0003.tif RecG complementation.

Figure S4 mbio.02156-23-s0004.tif RecG peroxide.

ASM does not own the copyrights to Supplemental Material that may be linked to, or accessed through, an article. The authors have granted ASM a non-exclusive, world-wide license to publish the Supplemental Material files. Please contact the corresponding author directly for reuse.
