# Supplementary figures and images for: The contribution of DNA repair pathways to Staphylococcus aureus fitness and fidelity during nitric oxide stress
Source: mBio. 2023 Nov 10;14(6):e02156-23. doi: 10.1128/mbio.02156-23 (PMC10746251; doi:10.1128/mbio.02156-23)

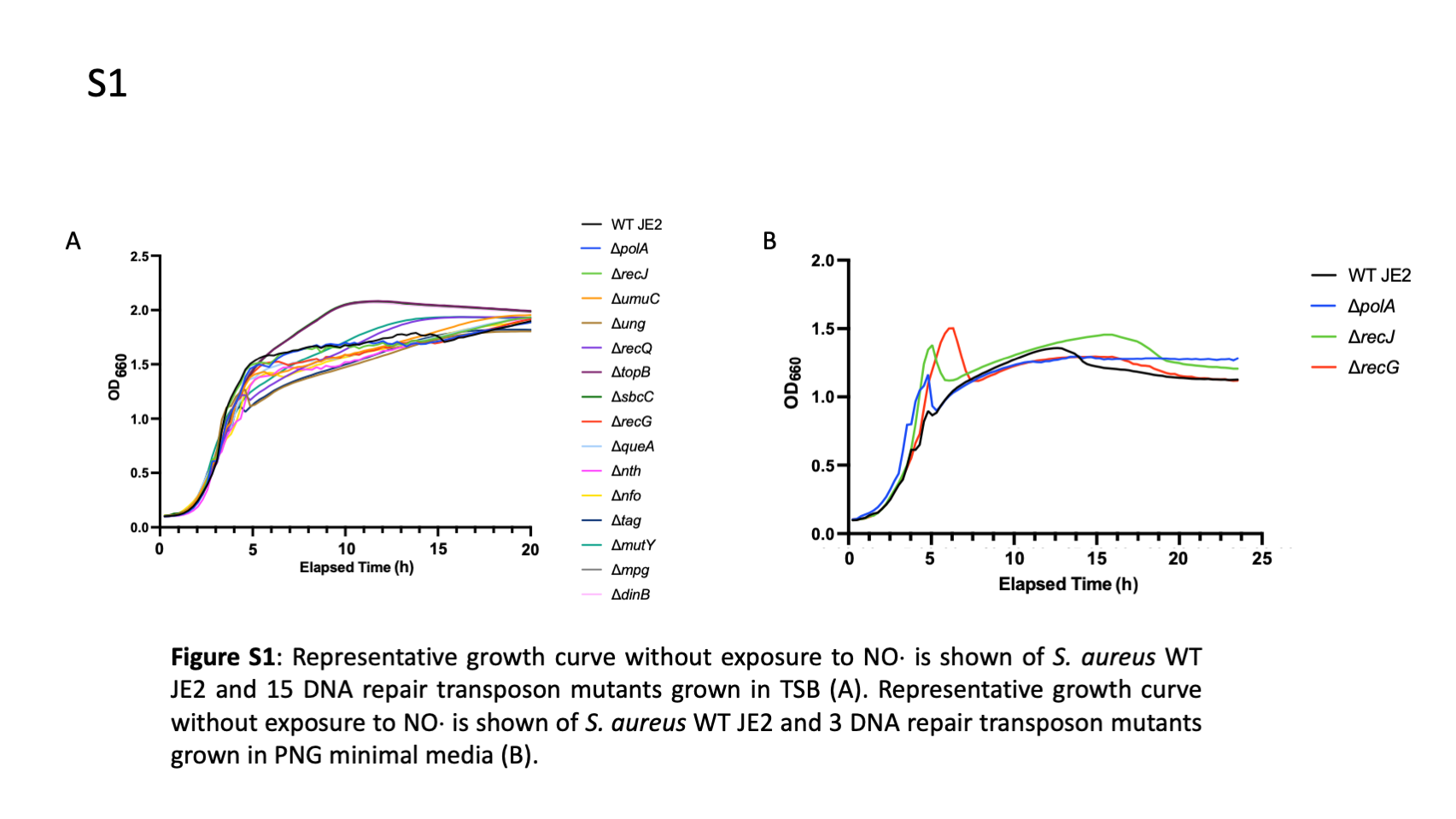

Supplement: Figure S1 — Growth without NO. [file mbio.02156-23-s0001.tif]

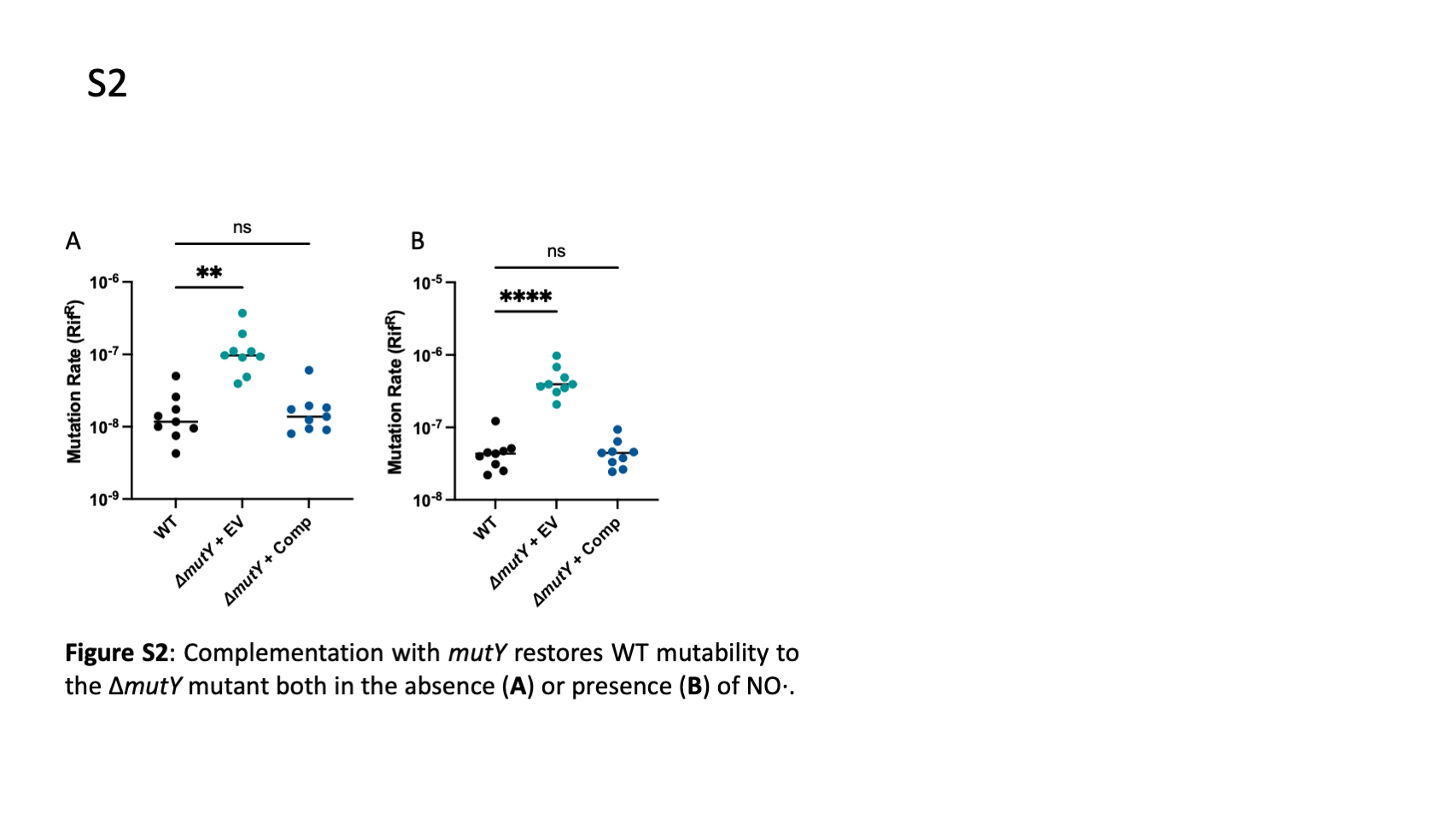

Supplement: Figure S2 — MutY complementation. [file mbio.02156-23-s0002.tif]

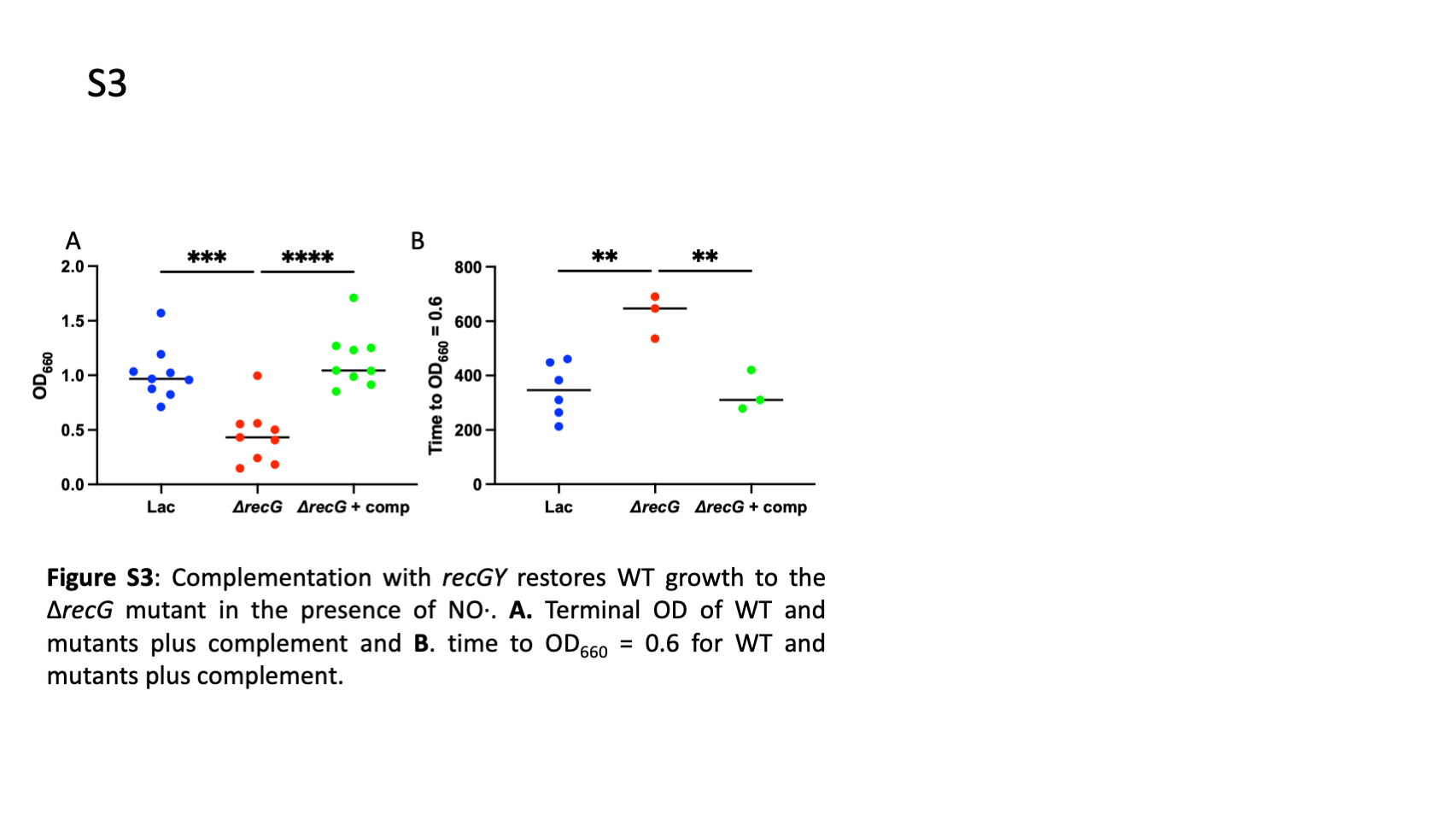

Supplement: Figure S3 — RecG complementation. [file mbio.02156-23-s0003.tif]

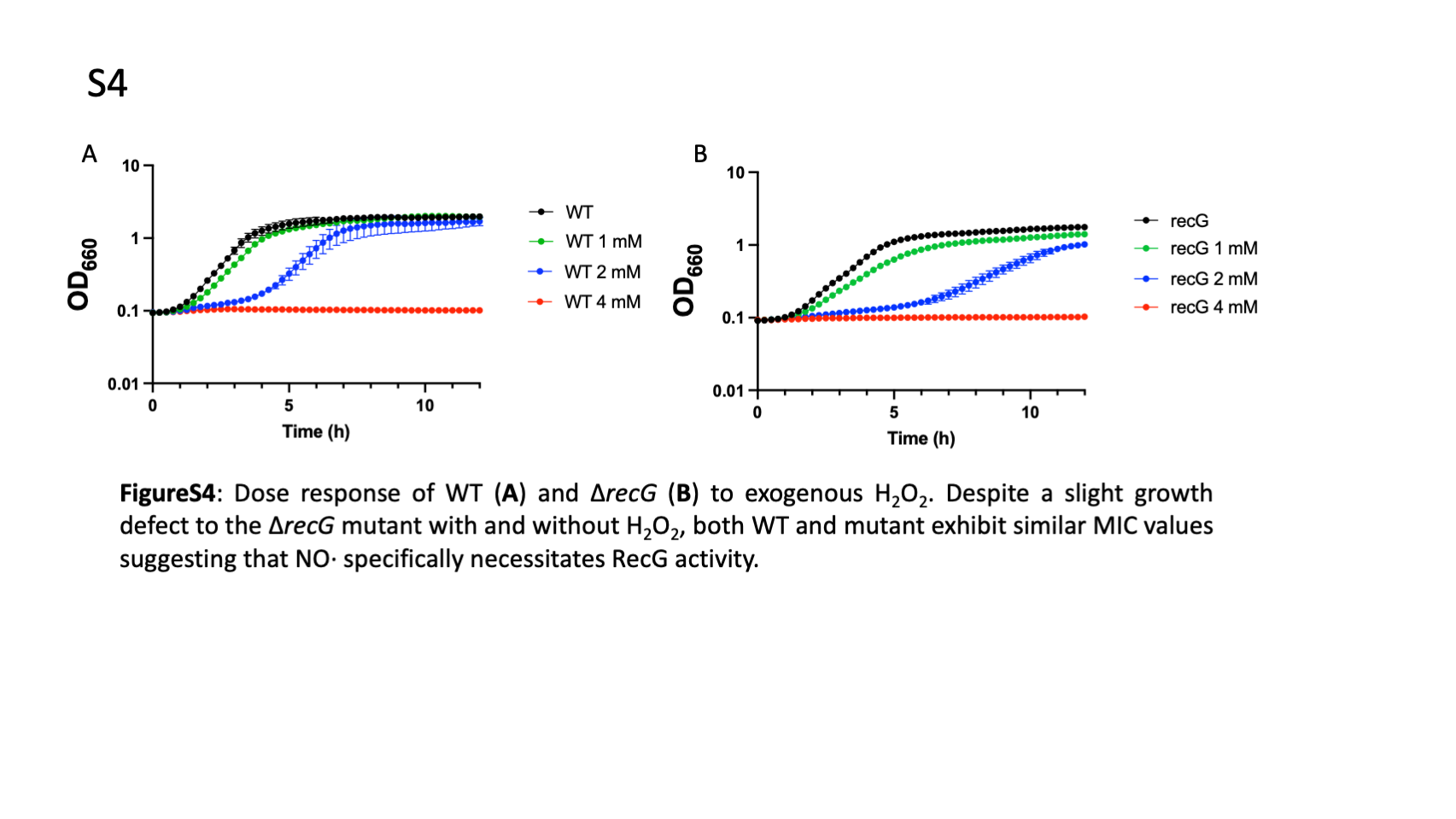

Supplement: Figure S4 — RecG peroxide. [file mbio.02156-23-s0004.tif]
